# Devices and Technology in Transcranial Magnetic Stimulation: A Systematic Review

**DOI:** 10.3390/brainsci12091218

**Published:** 2022-09-09

**Authors:** Mario Ibrahin Gutierrez, Irais Poblete-Naredo, Jorge Airy Mercado-Gutierrez, Cinthya Lourdes Toledo-Peral, Jimena Quinzaños-Fresnedo, Oscar Yanez-Suarez, Josefina Gutierrez-Martinez

**Affiliations:** 1 Subdirección de Investigación Tecnológica, División de Investigación en Ingeniería Médica, CONACYT —Instituto Nacional de Rehabilitación LGII, Mexico City 14389, Mexico; 2 Departamento de Toxicología, Cinvestav—IPN, Mexico City 07360, Mexico; 3 Subdirección de Investigación Tecnológica, División de Investigación en Ingeniería Médica, Instituto Nacional de Rehabilitación LGII, Mexico City 14389, Mexico; 4 División de Rehabilitación Neurológica, Instituto Nacional de Rehabilitación LGII, Mexico City 14389, Mexico; 5 Neuroimaging Research Laboratory, Electrical Engineering Department, Universidad Autonoma Metropolitana Unidad Iztapalapa, Mexico City 14389, Mexico

**Keywords:** transcranial magnetic stimulation, TMS technology, TMS coil, TMS stimulator, TMS devices, TMS modeling

## Abstract

The technology for transcranial magnetic stimulation (TMS) has significantly changed over the years, with important improvements in the signal generators, the coils, the positioning systems, and the software for modeling, optimization, and therapy planning. In this systematic literature review (SLR), the evolution of each component of TMS technology is presented and analyzed to assess the limitations to overcome. This SLR was carried out following the PRISMA 2020 statement. Published articles of TMS were searched for in four databases (Web of Science, PubMed, Scopus, IEEE). Conference papers and other reviews were excluded. Records were filtered using terms about TMS technology with a semi-automatic software; articles that did not present new technology developments were excluded manually. After this screening, 101 records were included, with 19 articles proposing new stimulator designs (18.8%), 46 presenting or adapting coils (45.5%), 18 proposing systems for coil placement (17.8%), and 43 implementing algorithms for coil optimization (42.6%). The articles were blindly classified by the authors to reduce the risk of bias. However, our results could have been influenced by our research interests, which would affect conclusions for applications in psychiatric and neurological diseases. Our analysis indicates that more emphasis should be placed on optimizing the current technology with a special focus on the experimental validation of models. With this review, we expect to establish the base for future TMS technological developments.

## 1. Introduction

The generation of a magnetic field produced by a wire conducting an electrical current was discovered by Hans Christian Oersted in 1819. In modern research, the first proposal of using a magnetic field to stimulate the brain was made in 1985 by Baker et al. [1] in a paper that presented a transcranial magnetic device for brain stimulation based on a circular coil placed over the head. This group had previously worked on transcranial electric stimulation (TES) with the objective of finding therapeutic techniques for neurological diseases [1]. After their partial success in that field, they continued working with the electric stimulation of the brain, instead producing the electricity by means of magnetic induction, which resulted in the development of the first formal proposal of a device that created a magnetic field to provoke a physiological effect in the brain [2]. Other studies have accompanied that first proposal by adapting the coils, improving the shapes, or proposing complex designs for specific purposes [3,4].

Just a few years after Barker et al.’s paper, a widely used coil with the shape of a number eight (now called figure-of-eight coil, i.e., a FoE coil) was used with success [3], and it became a useful tool in functional brain mapping (FBM) and motor-evoked potentials (MEP) generation. This new design and application allowed researchers to increase knowledge about the localization of motor zones in the brain, which was not fully accomplished with TES [2], because of the discomfort this technique produced in awake subjects. On the other hand, the near-zero amount of discomfort of TMS was useful for a wider spectrum of applications, where FBM [3,5,6] and evaluating the perioperative spinal cord function by MEP generation [2] were two of the most meaningful ones. In recent years, this line of research has extended to the assessment of neuroplasticity with TMS for certain diseases [7,8,9], the TMS monitoring of celiac patients [10], and the use of TMS as a diagnostic tool for study the central motor pathways [11,12].

The first TMS researchers used this technique to determine the cortical regions where the motor paths were located [3,5,6]. Certainly, most of the brain motor regions were discovered with this technique. Later on, TMS was gradually applied as a mean of therapy for psychiatric problems such as depression [13,14,15,16], bipolar disorder [15,17], and schizophrenia [18], among others. Moreover, TMS therapeutic capabilities were tested with success in neurological disorders such as Parkinson’s disease [19], Alzheimer’s disease [20], dementia [21], sleep disorders and insomnia [22], stroke [23,24], and traumatic brain injuries [25,26].

When this technology was initially applied to brain therapy, there was a concern about the effect of the repetitive pulses of TMS on the brain and its potential therapeutic benefits. Initially, TMS devices for brain mapping required short, but important, periods of time to recover before producing the next pulse. However, for therapeutic TMS, faster devices with shorter recovery times were designed for repetitive TMS (rTMS), which was proposed for the treatment of a wide variety of health problems [18,21,22,27,28]. Recently, theta-burst stimulation (TBS), another modality of TMS, has proved to be as effective as “classical” TMS, with shorter therapy sessions [29,30] for promoting neuroplastic effects in patients with cerebral diseases and trauma. Some studies have shown that the after-effects of TBS can last from minutes to hours [31], which produced strong inhibition of cortical excitability due to long-term depression-like changes in synaptic transmission [32,33]. Both functional and structural neuroplasticity have been observed after single and repetitive sessions of TBS [31,34].

**Figure 1:**
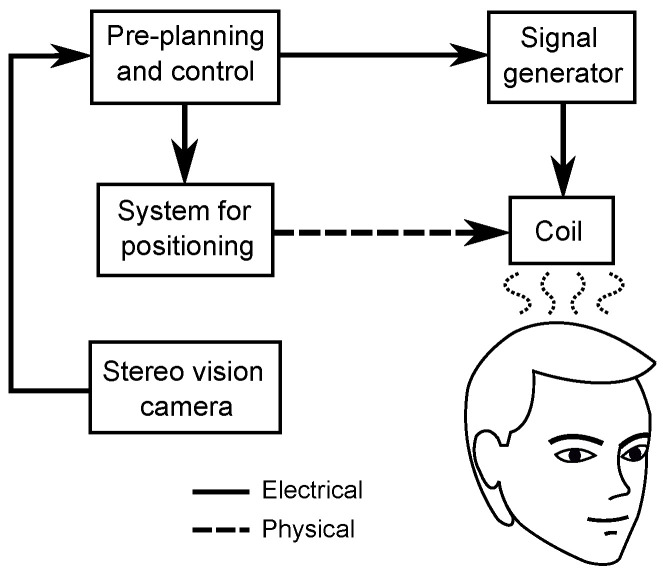
Block diagram of main components of TMS treatments.

In this systematic literature review (SLR), the technology for TMS (and its variations) is detailed and analyzed, with special emphasis on the signal generators, coils, positioning systems, and modeling approaches for coil optimization and therapy planning (based on the system of Figure 1). This study incorporates and describes the technological modifications of the TMS components for providing the readers a wider and deeper perspective of the state-of-the-art in TMS so they are able to choose the more suitable components for their intended applications or even to propose new designs for their own systems. The analysis carried out in this manuscript also provides the common applications of the technology that have been developed for TMS with the advantages and limitations of each configuration.

## 2. Materials and Methods

### 2.1. Search Strategy

A comprehensive search was carried out across four of the most important databases of research articles from April to June 2022: PubMed (National Center for Biotechnology Information, NCBI, Bethesda, MD, USA), Web of Science (WoS, Clarivate Analytics PLC, Philadelphia, PA, USA), Scopus (Elsevier, Amsterdam, The Netherlands), and IEEE Xplore (IEEE, Piscataway, NJ, USA). Records from conferences and other reviews were excluded from the initial search. All records found since 1985, which is the year of the first proposal of TMS, were included [1]. Indexes were downloaded by searching for the term “Transcranial Magnetic Stimulation” in the title/abstract; the acronyms “TMS”, “rTMS”, and variants were not used, since they retrieved a significant amount of records of unrelated topics. Data were exported to RIS and CVS format files depending on the database capabilities. Records were imported to the web application rayyan.ai for filtering and classification [35]. Additionally, a brief report of the number of world-wide patents in TMS was included, in order to give a timestamp of the state-of-the-technique; these were searched in Espacenet (European Patent Office) and WIPO IP Portal (World Intellectual Property Organization, Switzerland).

### 2.2. Research Questions

The objectives of this SLR are summarized within the next set of research questions to discuss the technological advances in TMS in more detail:

RQ1: Has the technology for TMS reached convergence?

RQ2: Is there a final solution for improvement assessment with TMS?

RQ3: Has the FoE coil been significantly improved over the years?

RQ4: Have researchers presented a definitive solution for TMS positioning systems?

RQ5: Have recent modeling software permitted to optimize TMS coils and procedures?

### 2.3. Inclusion and Exclusion Criteria

Records were filtered automatically according to the following terms about the technology for TMS: coil(s), device(s), stimulator(s), modeling, simulation(s), and computation(s); articles in other languages than English or Spanish were excluded. After that automatic selection, the remaining records were manually filtered by reading the title and abstract; articles that were not about testing, proposing, presenting, and studying components or systems of TMS technology were excluded. Other records that were not detected automatically with the previous criteria (conferences, reviews, and articles not excluded with the filtering terms) were also excluded in this second pass.

The remaining papers were retrieved and fully read. Articles with systems and techniques previously presented were excluded unless significant adaptations were proposed. Works about TMS systems for cell experiments or animal studies were excluded. Articles about clinical trials and case reports were also excluded, unless the device or technique used in the experiments was being presented for the first time. During the reading process, other papers were identified and included if they passed the inclusion criteria explained above. This procedure was based on the guidelines of the PRISMA 2020 Statement [36]. The authors of this SLR carried out this process blindly to reduce the risk of selection errors and selection bias.

### 2.4. Data Extraction and Analysis

In order to have an organized analysis of the selected records, these were grouped into four categories: articles presenting or adapting electronic circuits for coil stimulation, those proposing new or modified designs of coils, documents with proposals and systems for coil positioning, and articles presenting computational models of coils and optimization algorithms. Some records contributed to more than one category. Data analyzed in this review were extracted from the manuscript; some missing information, e.g., software used for modeling or devices used during the procedures, was obtained from other publications by the same authors. Records were classified and detailed in a spreadsheet, which can be downloaded as supplementary material. A simplified version of this information, including the records published during the last 5 years, is shown in Table 1.

## 3. Results

Devices used to generate high-power electrical signals for TMS have increased in complexity and capabilities but decreased in size and cost. The improved control systems warrant the adaptation of additional stimulation parameters that may modify the treatment outcome, which has allowed researchers to establish interrelations among TMS variables and observed therapeutic effects. In this review, we analyze the technology for TMS using four aspects: the devices for signal generation, the coils, the positioning systems, and the software used for coil optimization and therapy planning. This study was carried out following the recommendations of the PRISMA 2020 statement.

### 3.1. Study Selection

**Figure 2:**
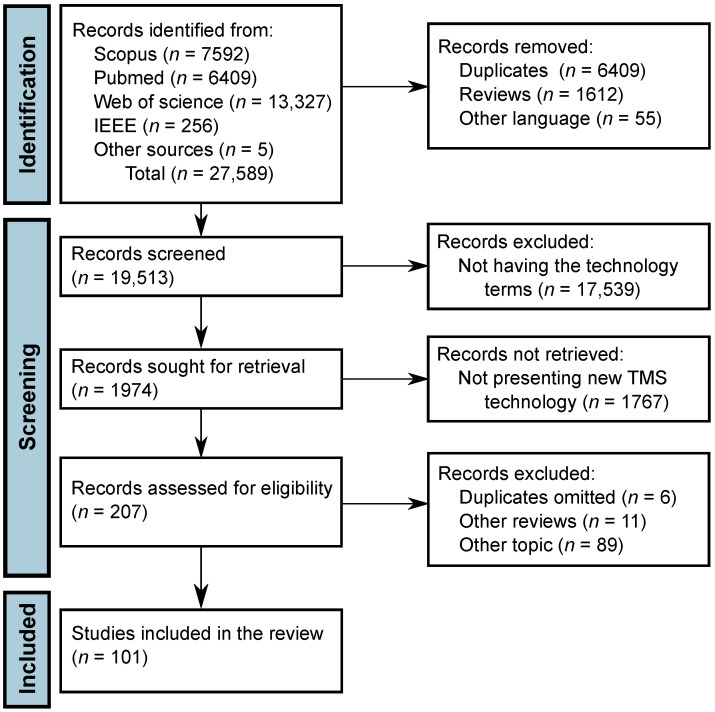
Flowchart of the Systematic Literature Review based on the PRISMA 2020 statement [36].

Based on the search criteria, there were 6409 articles in PubMed, 13,327 in WoS, 7592 in Scopus, 256 in IEEE Xplore, and 5 records from other sources, giving a total of 27,589. Among them, 6409 were duplicates, 1612 review papers, and 55 in a language other than English or Spanish. From the remaining 19,513, 1974 records were filtered using the specified terms about technology for TMS, and, after reading the titles and abstracts, 207 articles were selected for retrieval to be read carefully. Publications that did not present new technological developments in TMS were excluded, giving a total of 101 records that were included in this SLR. The included records from other sources were found from the references of the read papers or were proposed by experts in the field that read our manuscript prior to publication. This process is shown in Figure 2 [36].

**Figure 3:**
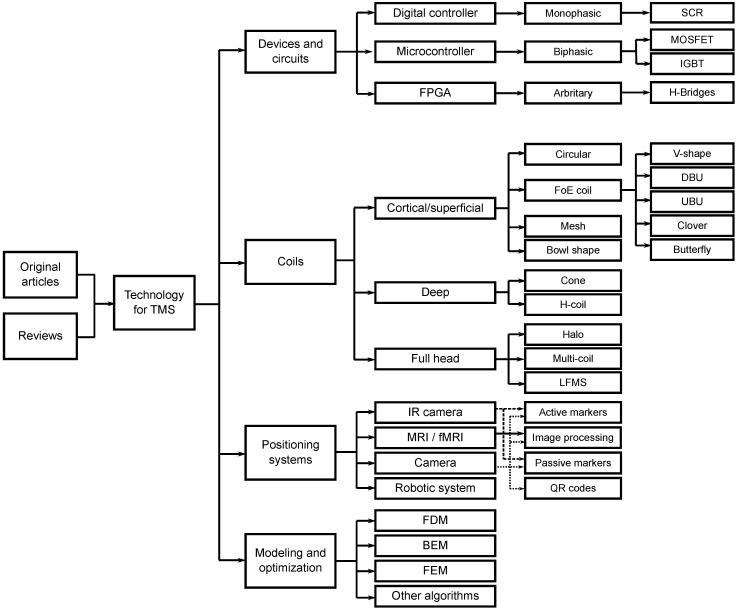
Taxonomy of TMS technology of this Systematic Literature Review.

These papers were classified into four categories: devices, coils, positioning systems, modeling and optimization; articles could be placed in one or multiple categories (see TMS taxonomy in Figure 3). From the included articles, 19 present devices, circuits, or optimizations of electrical parameters (18.8%); 46 show new coils or improve pre-existent coils (45.5%); 18 propose systems for coil positioning or therapy planning (17.8%); and 43 present models or propose optimization algorithms for coil design (42.6%).

Other reviews were also studied to contrast their contribution with our approach and topic. From the 1612 initially identified, 101 had the required filtering terms mentioned in the first paragraph (6.3%). After reading the titles and abstracts, 4 were identified as possible reviews of TMS technology. However, after retrieval and carefully reading, it was determined that only one record could be (marginally) used to compare our work. By consulting other sources, we found 1 book chapter about this topic that can be used for comparison [73]. This process was also carried out with rayyan.ai [35].

World-wide patents of TMS were consulted to determine a 2022 timestamp of the state-of-the-technique. However, full-text documents were not retrieved, since that is beyond the scope of this study. Using the terms “transcranial magnetic stimulation” in the title and abstract in the database Espacenet, we obtained 596 results. After a more specific search including the filtering terms used for the research articles (coil, device, and stimulator), the records were reduced to 291. Using the same initial terms (“transcranial magnetic stimulation”) in WIPO IP Portal, 5243 records were obtained.

### 3.2. Stimulators

The technology of TMS stimulators has improved over the years, with the use of different high-power solid-state devices and coil configurations. Many levels of optimization have permitted improvements in efficiency that have opened up the possibility of more complex pulse excitation protocols, with the capability of adjusting the repetition frequency, waveform, etc. In order to accomplish the required magnetic field of about 2 T [74], the excitation voltage and current of the coil could reach about 10 kV and 10 kA, respectively. The combination of these large excitation parameters with low coil resistance produces important heating and acoustic noises in the coil system during the session that can have an impact on the therapy outcome [66]. Increasing the efficiency of the whole device would directly permit the reduction of coil heating, with minimal attenuation of the acoustic noise; nevertheless, this noise still represents a key factor in addressing future developments [75].

**Figure 4:**
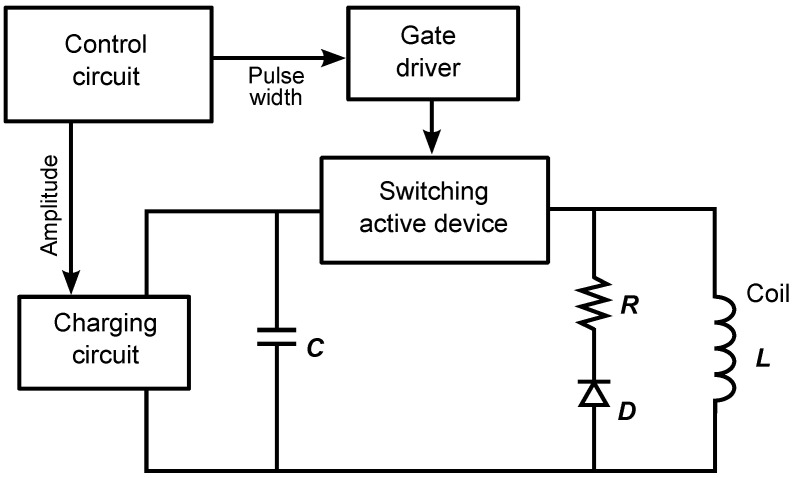
Basic circuit of TMS stimulator.

The electrical signal generator for TMS coils is usually composed of three basic components, as shown in Figure 4, namely, a capacitor (*C*) for energy storage, a switch to temporally connect the coil to the capacitor (usually a silicon controlled rectifier, SCR), and a high voltage source to load the capacitor when it is not connected to the coil. The practical TMS coil can be represented by an inductance (*L*) in series with a small resistance (*R*). The three *RLC* components harmonically oscillate to produce a decaying sine-shape electrical current that produces a magnetic field when passing through the coil. Other components are usually required to keep the circuit working and make it able to operate as expected, for instance, capacitors, diodes, transformers, resistors, and control circuits. The final configuration of these main components can be used to adapt the shape and amplitude of the electrical current and other parameters such as pulse duration and repetition frequency (for repetitive TMS).

Despite the simpleness of the basic stimulator circuit for TMS, it can still be subject not only to optimization, but also to improvement. In 2005, Davey and Riehl proposed the optimization of a TMS system by identifying the optimal parameters of both circuits and coils [76]. They implemented a combination of numerical techniques to analyze the magnetic field produced by the coil (by varying the number of turns and coil size), the voltages and currents in the electrical circuit for different components’ values, and the effect of core saturation using non-linear analysis. Burunkaya [77] proposed incorporating a dsPIC (digital signal Programmable Intelligent Computer, Microchip Technology Inc., Chandler, AZ, USA) into a SCR-based TMS device in order to control the charge and discharge of the capacitor and to include the possibility to repeat the stimulus with a programmable repetition frequency. The main proposal was to replace, with a microcontroller, the control stages usually carried out with a computer. Although the idea was adequate at the time, the use of computers for other purposes, such as magnetic resonance imaging (MRI) segmentation, therapy planning, and real-time coil positioning, would make this replacement unnecessary.

Other improvements were applied within the incorporation of insulated gate bipolar transistors (IGBTs) and metal–oxide–semiconductor field-effect transistor (MOSFET) in fast-switching high-current applications and low-cost low-current devices, respectively [78]. Peterchev et al. proposed to replace SCRs with IGBTs to actively control the *on–off* of the switching device to modify the duration of the excitation signal [79]; a SCR can be turned *on* with a pulse at the gate terminal, but it is turned *off* by itself when the current between its terminals reaches zero. IGBTs can be used to control the pulse width by intentionally turning them *on–off* with an adequate square voltage at the gate terminal. Based on this idea, Gattinger et al. proposed an H-bridge circuit with IGBTs capable of producing different types of excitation, from monophasic to biphasic pulses, and even sinusoidal or quasi-squared excitation [80]. Similarly, Ha et al. proposed a three-stage bridge circuit to control the pulse shape and repetition frequency of the excitation signal by means of a microcontroller [81]. Recently, this was taken to a more complex design by Zeng et al. [72], with the proposal of a IGBT-based modular system that produces digitally-controlled arbitrary current waveforms with reduced sound at the TMS coil.

The use of multichannel excitation systems was a logical step after the proposal of coil arrays [82] and meshed configurations [83]. Most of these applications are composed of *N* independent stimulators synchronized by a common digital controller [84]. However, even when using independent signal-generation devices, the electromagnetic coupling among adjacent coils is strong enough to affect other drivers [85]. Active devices used for TMS stimulators require certain voltages and currents at their terminals to effectively switch at the required speed. When the load is not fully passive, the induction of backward currents due to external interference (such as magnetic couplings) provokes variations in the output waveform, which reduces the capability to control the device commutation. Due to the fact that this is still subject to study, it is possible to provide TMS multichannel stimulators with enhanced features, such as the one proposed by Xiong et al. [39,86], which included systems for data acquisition (DAQ) and sensors for 3D magnetic field measuring; all of them were controlled by an FPGA (field-programmable gate array).

Innovations have been made in other components of the TMS system. In 2010, de Sauvage et al. designed a portable TMS system based on SCR [27]. The system proved to be lightweight and capable of producing current densities of 1.9 times the motor threshold. Later, Peterchev et al. [66,75] proposed to reduce the loud sound provoked by the coil during therapies by implementing a double plastic case around the coil to absorb the acoustic waves and reduce the undesired effects that these sounds could have on the therapy. Other groups have proposed adaptations of the TMS devices and coils to provide double-blind sham stimulation [87] by controlling the direction of the electrical current in the coils to deliver either sham or effective (real) therapy [71].

### 3.3. Coils

**Figure 5:**
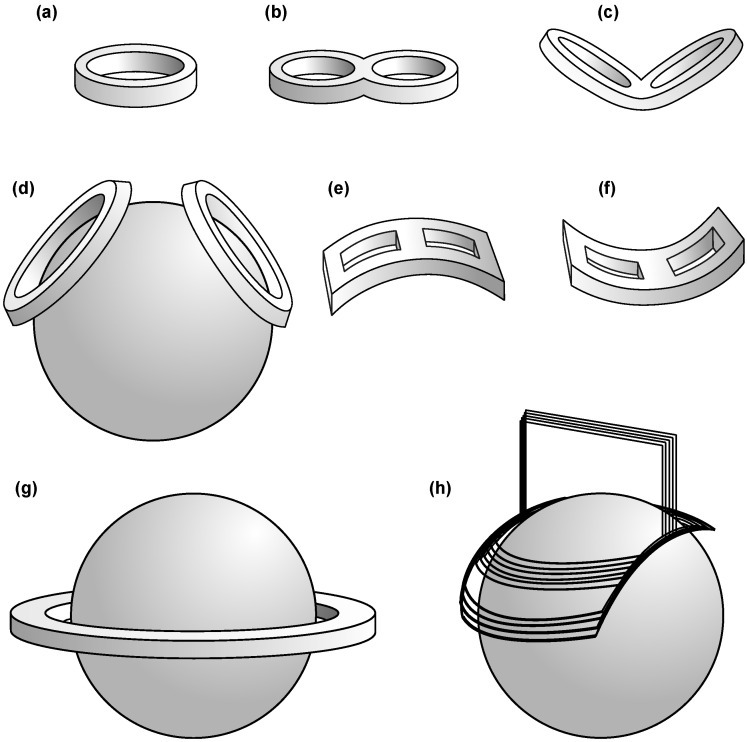
Most used TMS coils. (**a**) Circular coil, (**b**) Figure-of-Eight (FoE) coil, (**c**) Butterfly (V-shape) coil, (**d**) Cone (double circular) coil, (**e**) Downward-Bending U-shape (DBU) coil, (**f**) Upward-Bending U-shape (UBU) coil, (**g**) Halo coil, and (**h**) Hesed coil (H-coil).

Since the first paper of TMS was published in 1985 [1], different coils have been proposed to improve the magnetic field concentrations in certain brain regions [88], some of them shown in Figure 5. The first work was carried out with a circular coil that produced a non-focused rounded field that corresponded to the shape of the stimulation coil. A year after that, Merton and Morton proposed a twin array of circular coils for TMS [89], and, independently, Ueno et al. proposed a similar array for the precise excitation of a small cortical brain region, which further permitted them to functionally map the brain [3]. Since then, research groups and manufacturers have proposed many coils for different applications [57], some of them being variations of these two initial developments. These were compared in 2013 by Deng et al. in an interesting article that included 50 coils for TMS [88].

One of the most used coils for TMS is the FoE coil (figure-of-eight coil, also referred to as the double circular coil [89] and butterfly coil [90]). This particular configuration is composed of two circular coils of about 5 cm to 15 cm in diameter, either very close to each other or overlapped at their intersection [91]. The electrical current moves in the opposite direction at each circular coil, which produces a convergent induced electric field at the intersection [92]. If the direction of the current is the same in both coils, the induced electric field at the center would theoretically be zero, which allows for the production of sham TMS with the same FoE coil by changing the electric current direction in one of the circular coils [93]. This coil is useful when the TMS is required at small superficial cortical regions (about 5 mm), for instance, in FBM, for producing MEP, and for the treatment of some psychiatric disorders [94].

In 1993, Kraus et al. adapted the circular coil with a curved shape that mimics the curvature of the head to reduce positioning problems and variability in therapy results obtained with conventional circular and FoE coils [2]. Although the penetration depth was improved, the larger brain volume stimulated with this coil could produce confusing results due to simultaneous multiple muscle responses [95]. Therefore, based on its limited focality, this coil is not useful for functional brain mapping. However, this multi-muscle activation could be an advantage under certain circumstances, for instance, in the perioperative fast evaluation of the spinal cord function. The use of cap-shaped coils for therapeutic TMS applications should not be discarded.

The inclusion of magnetic materials in the core could improve the efficiency of the coil in generating the magnetic field. Epstein and Davey in 2002 implemented an iron-core FoE coil to concentrate the magnetic flow and increase focality [96]. Their coil induced a more intense electric field with less temperature increase than the air-core coil of the same size. However, this design was not effectively implemented by others due to concerns of producing important eddy currents, increased heat in the core, or even lower efficiencies for high repetition rates due to core saturation. Along the same path, other designs using iron-core materials and windowed shielding plates with improved focality [97,98] and water-cooling stimulation (WCS) coils [60] have been also proposed. Applications of these coils designs are those detailed for the FoE coil.

The use of a metal shield with a window to reduce the magnetic field at zones out of the region-of-interest (ROI) was first proposed in 2006 [99]. For this, a metal plate was placed between the coil and the subject at varying distances. The plate was made of copper. A customized window was opened at the middle of the coil, with the objective to only let = the magnetic field at the center of the FoE coil pass. Many analyses were carried out with different window sizes, varying distances among the components of the system (the coil, the shield, and the subject) [99], and different magnetic conductive materials at the back of the coil [100], among others. This design provided a more controlled dose to the treated zone with increased focality, less lateral stimulation, and higher efficiency. However, conductive shields reduced the intensity of the field in the ROI compared with no-shield condition, which should be taken into account.

In 2018, a semi-ellipse downward-curved FoE coil (also referred to as Downward-Bending U-shaped coil [61]) was proposed [43]. This coil is a modified FoE coil, downward-curved to follow the shape of the head to improve focalization and intensity at the treated zone. However, based on the results, although the bending improved the intensity of the induced electric field, it reduced the focality for certain angles compared with the FoE coil. Two years later, this group improved the focality by bending the coil upwards (Upward-Bending U-shape (UBU) coil) to reduce the effect of the winding. A similar proposal was made by Eaton in 1992 [101] using a V-shape coil with not fully satisfactory results, probably because the contact point to the head was small compared with the UBU coil proposed by Fang et al [61]. Having a U-shape instead of a V-shape coil provided a wider contact zone to the head that increased the delivered magnetic field.

This type of upward oriented/bent coil is called a butterfly coil. Some groups have proposed the use of double butterfly coils [90], also called quadruple butterfly coils [102,103], of different sizes to improve focality and reduce the lateral lobes where the “wings” are usually placed in FoE coils. Using this modified version of V-shape coil, the focality in the ROI was improved as expected. However, the intensity of the magnetic flux at the ROI decreased with the double butterfly coil compared with the FoE coil [102]. This can be improved by incorporating additional passive shielding materials with certain shapes close to the focalization zone [44]. The use of a double upward–downward butterfly coil has proven to produce larger focality compared with the FoE coil under similar conditions [69]. Applications of this new coil range from the same mentioned for the FoE coils (FBM, MEP, rehabilitation, etc.) to any new proposal that requires superficial TMS for either therapy or improvement assessment [8].

Regarding optimization, other parameters have improved the field intensity and focality. The coils could be symmetrically designed (concentric), with the turns equally spaced from the center, or spaced asymmetrically, with its center displaced from the circumference (eccentric), or with variations in the winding shape to reduce the Lorentz forces [59]. Applying this variable to the FoE coil, it is possible to obtain significant differences among measurements of the two mentioned configurations [104], the latter being more efficient. Other parameters can be adapted for optimizing the field and reducing lateral lobes, as the design proposed by Koponen et al. that was based on the FoE coil, by instead extending it to cover the head [105]. Li et al. [106] modified the spacing and length of wire turns of a FoE coil, with discrete improvement. Moreover, the use of a computational framework based on the inverse boundary element method (IBEM) to analyze coils and to obtain optimized configurations from an initial design was recently proposed [40]. The use of a full-head optimized FoE coil limits its practicality, since it can not be placed at any region of the head; therefore, the potential applications of these coils are in the field of repetitive therapy for psychiatric diseases and neurological problems.

Although the FoE coil has demonstrated its applicability for focused TMS with few required improvement and optimization [88], it is not very useful when the therapy protocol requires deeper or full-head stimulation. This could be partially solved by using an array of circular coils together providing a complex magnetic stimulation of the cortical regions [42,70,82,107,108] with capabilities of multiple stimulation patterns [62] and complex optimization algorithms [108]. Although the endeavor of placing a large number of coils is not an important factor to consider if they are fixed in a chassis, the determination of their effects after superposing the fields produced by these coils is not simple. Other options for deep-brain TMS are the cone coil and double cone (also called biconical or twin circular coils), which could produce deeper stimulations controllable with their respective angles [46,91,109]. Moreover, a bowl-shaped coil, intended for superficial TMS, can make the treated region significantly wider [98]. However, more full-head designs with more controllable capabilities would allow deeper magnetic stimulation with reduced effects at surface regions.

In 1992, Roth et al. proposed the Hesed coil (also called H-coil) to be able to produce a focalization zone into the head [4]. This coil is composed of a complex winding that covers all the head [41] or a part of it, depending on the protocol [110]. The design of the coil winding is made numerically in order to produce a summation of the electric field at a certain brain region. This coil can be customized to produce focalized stimulation in deep regions of the brain with little effect in cortical zones; however, it still required more work to fully determine its practical usefulness in clinical practice. After the first proposal in 2002, many variations of the first design have been suggested, for different deep TMS applications [111], these being the therapy of psychiatric and neurological diseases the most common [112,113].

The use of a mesh of wires placed as a cap over the head was proposed in 2013 by Jiang et al. [114]. This mesh can be driven using a multi-channel system that would inject the electrical current into individual wires to form a customized shape of excitation, including the shape of classical circular and FoE coils. Although this development produces the equivalent effect of different types of coils [83], it is still under research because of the technical difficulties associated with a multichannel stimulator capable of driving this configuration. Having multiple coils placed very close to each other produces mutual inductances that modify the design parameters and may affect the operation of the excitation circuit [85]. Potential applications of this technology include functional brain mapping, the generation of motor evoked potentials, and therapy for psychiatric and neurological diseases.

Another solution for deep-head stimulation is the Halo coil [115], which is usually combined with other smaller coils (as circular [115,116], FoE [54], etc.) to achieve certain desired stimulation patterns. This coil is an adaptation of a circular coil but with increased size, capable of being placed around the head. The magnetic field at the center of the coil enables the stimulation of the central regions of the brain. Arrays of 3 Halo coils further provide multi-center configurable stimulation protocols, with limited superficial excitation. Other authors have proposed more complicated systems for full-head therapy in low-field magnetic stimulation (LFMS) with adequate full-head uniform distributions [47]. Common applications of these full-head and deep-brain proposals are in line with the therapies used in psychiatry and neurology.

Finally, because TMS must adapt to therapy and research requirements, additional technology for TMS testing and evaluation has been developed. In order to assess the TMS efficacy in patients, MRI-compatible and sham coils have been designed. In 2015, Navarro et al. proposed a coil for combined TMS/fMRI (functional MRI) experiments [117]. Three years later, Lu and Wang proposed a design with two concentric circular coils for TMS and MRI applications to be used separately [37]. In 2006, Sommer et al. presented a method for sham stimulation by combining two FoE coils (a sandwich coil design), with only one being active [118]; sham stimulation was delivered when the non-active coil was touching the head, keeping away the active coil. A similar approach was proposed by Rossi et al. a year later, which consisted of one FoE coil and wooden material to physically separate the coil from the head [28]. Years later, Takano et al. proposed a modified coil with internal electric connections that reduces the magnetic field, by destructive interference, for sham stimulation [65].

### 3.4. Positioning

A very important part of the application of TMS is the correct and accurate coil positioning over the ROI in the head, either for assessment or therapy applications. This task is usually accomplished "by hand" with the guidance of protocols based on direct measurements with reference to head landmarks [49,119], with the assistance of surgical tools [120] or even using MRI images and landmarks to manually locate the coil [121]; as can be expected, the success of these procedures is strongly influenced by operator expertise. Using mathematical and computational methods for coil positioning and orientation to maximize the delivered energy in the ROI is desirable, since it achieves repeatable results and increases therapy effectiveness [67].

In order to provide more precise positioning of the coil, camera-based infrared (IR) systems with 2D (stereo) [122] and 3D configurations [123] have been proposed. These consist of two or three cameras placed at a fixed location, which record the TMS treatment; sets of passive (IR reflectors [45,48], QR codes [123], etc.), or active (IR diodes [120]) markers are placed at the coil and the patient’s head to determine their real-time positions.

The use of MRI and fMRI allows the combination of the precision of those techniques for imaging with the capability of TMS to produce evoked potentials. Neggers et al. conceived a neural navigator (NeNa), a stereotaxic method validated with fMRI, and motor-evoked potentials for effectively positioning the TMS coil [124]. The results show precise control of the treated zone, but with complex preparation procedures. Another similar proposal was made by Herwig et al. [125] in which the rTMS was provided with the assistance of imaging the data of the positron emission tomography (PET) and a surgical tool navigator. The ability to reach the treatment zone was acceptable, but using PET data for this application would make this procedure more complicated.

Several groups propose the use of robotic systems combined with IR cameras to provide real-time localization of both the head and the coil in the space. The implementation of a robotic arm to hold and move the TMS coil coupled with IR passive markers and a commercial stereo camera is advised in different approaches [51,53,126,127]. This type of robotic arm provides a wide range of movement around the head and is suitable for different types of coils. Furthermore, custom-made systems for TMS, such as the robot-chair presented by Zorn et al. [128], enable a faster and controllable setup to be used routinely in clinic.

The regular and continuous use of TMS in hospitals requires short sessions with standardized procedures for the fast-positioning of the coils while maintaining the safety of patients and operators during the process. For this purpose, TMS systems should be robust with special emphasis on providing the auto-calibration of the electric sensors and the IR systems for positioning [45,64,129]. Although these robotic systems warrant the best performance for precise target localization, in many institutions, automated positioning systems are not the rule, but the exception, because of their relatively high cost and complex setup. More accessible and easy-to-use developments for TMS navigation are required, for instance, researchers creating their own developments based on open source initiatives [123].

### 3.5. Modeling and Optimization

Software is another important part of the technological development of TMS. In 1985, when the first TMS application was developed, computers were not widely used, and the software for modeling physical phenomena was not common. Nowadays, computers are more powerful and accessible to everyone, which allows us to employ simulation tools to improve and optimize TMS devices. The use of modular interfaces combining software and hardware components has also been proposed to standardize experiments and clinical trials [13,130]. These strategies, combined with more efficient devices, provide reliable and repeatable results in incoming studies.

Modeling approaches from recent years apply discretization paradigms, in which continuous domains are divided into small elements, where the calculations are made. Those methods are present in an important part of the literature on TMS modeling and are used to determine realistic electromagnetic distributions [58]. Publications about modeling and optimization in TMS can be grossly divided into three main branches, (1) modeling with the finite element method (FEM) [43,52,82,92,98,99,102,115,131,132], (2) modeling with the boundary element method (BEM) [133], and (3) modeling using the finite differences method (FDM) [42,58]. Adaptations of these procedures for fast computing and optimization, such as the improvement of BEM with the fast multipole method (BEM-FMM) [38,55] or the use of the inverse boundary element method for coil calculation [40,59,134], were presented. Algorithms adopting alternate programming languages for field optimization [42,56], fast field calculation with neural networks [63], or other analytical methods [107,135] are scarcely present. Reconstructed 3D models with MRI images and TMS [41,136], including temperature analyses [137], and incorporating experimental validation of TMS fields [68] are also presented.

## 4. Discussion

The reviewed papers were analyzed based on four classifications. These permitted us to adequately answer the research questions initially proposed in our study. The technological advances of the last two decades enabled researchers and engineers to improve the capabilities of the stimulation devices and TMS coils, with the help of new modeling strategies and computational systems. Although the evidence suggests that designs of devices for TMS, including coils, may have reached convergence (RQ1), technology is continuously advancing, so we could expect to have new proposals in TMS over the coming years. The necessity of validated therapy strategies that are fully effective for deep-brain TMS should encourage designers to propose further developments in this specific area. Coils with improved focality have been the objective of certain groups with promising results [69,100]; however, more emphasis on coil optimization should permit much better focality with smaller lateral lobes for FBM and MEP applications [44].

With these thoughts, we can conclude that there is not a final solution for TMS applied for the evaluation of motor cortical excitability using MEP (RQ2). Moreover, there are many paths with different approaches for evaluation using MEP [7,8,124]. The combination of TMS and other technologies for response registration (EEG, EMG) and diagnosis (fMRI) permits us to widen the possibilities. However, more controlled clinical research should be conducted to effectively determine the usefulness of this technique in relatively new proposals, for instance, in assessing neuroplasticity in patients undergoing rehabilitation [7,8], in the TMS monitoring of celiac patients [10], in the diagnosis with TMS of diseases affecting the central motor pathways [11,12]. Although the efficacy of using TMS for spinal cord evaluation with MEP has been widely recognized [3,5], the technology for this application is still improving [94].

TMS coils designs have also been improved in the last few years [88]. Coils for deep brain TMS have changed significantly, with complex multi-coil proposals being used in pulsed regimes (rTMS) [54]. Other designs produce a more distributed field over the head [4,111], either for full-head therapy or deep TMS applications. Focality in the FoE coil has been increased with the incorporation of multichannel excitation systems [39], coil arrays [62], magnetic materials for shielding [44,100], and mesh-based paradigms [83,114]. Implementing the coil and the positioning system in the same setup has reduced therapy durations [83]. Combined software–hardware architectures for parameter control and automatic data post-processing has permitted researchers to standardize TMS procedures [13,130]. Although the FoE coil has been importantly improved over the years (RQ3), its optimization is still required to have better focality, increase field intensity, and reduce coil and devices sizes [94].

The topic of robotic systems for positioning is still open for improvement (RQ4). Current robotic guiding systems [51] combine off-line head segmentation with real-time positioning using IR cameras [48], active markers [45], and image tags (QR codes) [53]. However, coil positioning is still limited by time-consuming calibration protocols to be carried out before each therapy [45], which prolongs the time required for each TMS session. Using autocalibration systems based on the same positioning devices used for monitoring (IR cameras, tags, markers) is still under research by some groups [45,64]. Simplification of these paradigms should be part of the priorities for improving TMS applications.

Optimization techniques and algorithms for coil design and therapy planning are growing fast. There are important aspects to consider when analyzing the modeling paradigms for TMS, for example, the required computer capabilities, the use of more reliable 3D modeling software based on medical images, and the required spatial/temporal resolutions of the final model. Current strategies propose optimization techniques for certain coils in order to provide increased focality with larger field intensities [69] by using geometry variations [98] and coils shielding (RQ5) [44,100]. The current most used approach for 3D modeling is based on the finite element method [43,53,88], which consumes large amounts of computational resources. Other numerical procedures for field modeling and coil designing have proven to be efficient when assuming simplified geometries [42,134]. However, more research is required within this line in order to experimentally validate new optimization approaches.

Future outcomes for TMS would be related to aspects of the modeling and optimization of current coils and the proposal of new coil designs with the help of modeling software. The singularity of different manufacturer coils is another aspect to uniformize [138], by means of comparative results [57], clinical-trials, and meta-analysis, in order to produce new, evidence-based, standards. The trade-off between focality and deep brain stimulation is another aspect to improve [139]. The design of new stimulation circuits is not discarded from future perspectives. Devices capable of producing adaptable wave forms, e.g., arbitrary wave generators for TMS, are still under development by some groups. Implantable magnetic stimulation devices are also under development for long-term applications [140]. Clinical applications of these new paradigms are going to be under research in the next few years.

## 5. Conclusions

TMS technology has evolved during the last few years. The development and usage of complex configurations of coils will enable researchers and clinicians to propose diverse strategies of brain stimulation and even to discover novel applications. The miniaturization of control systems, the incorporation of modeling techniques, and the optimization of TMS devices have improved the overall therapeutic effects of TMS. Even though TMS technology has advanced, we prospect further innovations to achieve more powerful and more precise magnetic stimulation of brain structures. The use of TMS for the assessment of motor responses and neuroplasticity is undergoing strong development in combination with other technologies such as EEG, EMG, and fMRI.

## Figures and Tables

**Table 1:** Records considered for this review of the last 5 years (2018–2022).

Author	Year	Active Device	Coils	Positioning Systems	Modeling and Optimization	Tested in Humans	Common Applications
Lu et al. [37]	2018	-	Circular	-	-	Yes (n=1)	TMS-fMRI
			Tuned for MRI				
Makarov et al. [38]	2018	-	FoE	-	BEM-FMM	No	Rehabilitation
					FEM		
Xiong et al. [39]	2018	-	Multicoil	-	-	No	Not discussed
			3 circular coils				
			3D coil sensor				
Cobos et al. [40]	2018	-	Rectangular	-	IBEM	No	Not discussed
			Double spherical				
			Hemispherical				
			FoE (centered)				
			FoE (non-centered)				
Fiocchi et al. [41]	2018	-	H-coil (dTMS H4)	-	FEM	No	Food addiction
			FoE				
Gomez et al. [42]	2018	-	FoE	-	Numerical	No	Not discussed
			Array circular coils		MRI images		
			Spherical coils				
Fang et al. [43]	2018	-	FoE semielipse	-	FEM	No	Exploratory
			FoE				
Rastogi et al. [44]	2018	-	FoE	-	FEM	No	Psychiatry
			Quad butterfly				
			Coil shielding				
Wang et al. [45]	2018	-	FoE	Robot arm	-	No	Not discussed
				Camera (stereo)			
				One-step calibration			
				Passive markers			
Wu et al. [46]	2018	-	Biconical	-	Numerical	No	Not discussed
Wang et al. [47]	2018	-	LFMS	-	FEM	No	Psychiatry
			Cap Coil		Numerical		
Ambrosini et al. [48]	2018	-	Double cone coil	StimTrack	-	Yes (n=19)	MEP
				Passive markers			
Trapp et al. [49]	2019	-	-	Wishbone	-	Yes (n=5)	Psychiatry
		**Device**		**Systems**	**Optimization**	**Humans**	**Applications**
Belyk et al. [50]	2019	-	FoE	-	-	No	Not discussed
		-	Cover				
Goetz et al. [51]	2019	-	FoE	Robot arm	-	Yes (n=21)	Rehabilitation
				Camera (stereo)			
				Passive markers			
Htet et al. [52]	2019	-	FoE	-	FEM	No	Not discussed
					BEM-FMM		
Lin et al. [53]	2019	-	Circular	Robot arm	-	Yes	Rehabilitation
				Monocular camera			
				QR tags			
Rastogi et al. [54]	2019	-	Triple Halo	-	FEM	No	Hippocampus
			FoE				and amygdala
			Array				stimulation
Makarov et al. [55]	2020	-	FoE	-	BEM-FMM	No	Rehabilitation
			Double FoE				
			Double cone coil				
			Double circular				
			3 axis coil				
Xiong et al. [56]	2020	-	Double layer array	-	-	No	Not discussed
Spampinato et al. [57]	2020	-	Double cone coil	-	-	Yes (n=13)	Cerebellar stim.
Gomez et al. [58]	2020	-	FoE	-	FEM	No	Not discussed
					FDM		
					BEM		
Cobos et al. [59]	2020	-	Double spherical	-	BEM	No	TMS-fMRI
			Hemispherical		IBEM		
			FoE (centered)				
			FoE (non-centered)				
Fang et al. [60]	2020	-	FoE with water	-	FEM	No	Exploratory
Fang et al. [61]	2020	-	FoE	-	FEM	No	Exploratory
			DBU				
			UBU				
Navarro et al. [62]	2021	-	3 axis coil	-	BEM-FMM	No	Not discussed
			FoE				
Sathi et al. [63]	2021	-	V-shape	-	Neural Network	No	Not discussed
			Halo coil				
			Array				
Noccaro et al. [64]	2021	-	FoE	Robot arm	-	Yes (n=6)	MEP
				Monocular camera			
				Passive markers			
Takano et al. [65]	2021	-	FoE	-	-	No	Psychiatry
			sham				
Koponen et al. [66]	2021	-	FoE	-	FEM	No	Not discussed
			Acoustic case				
Gomez et al. [67]	2021	-	FoE	Aux dipole method	-	No	Not discussed
				Open source			
Afuwape et al. [68]	2021	-	Double FoE	-	FEM	No	Not discussed
			Quad butterfly				
			Triple Halo				
		**Device**		**Systems**	**Optimization**	**Humans**	**Applications**
Zhang et al. [69]	2021	IBGT	Circular	-	-	No	Not discussed
			FoE				
			Double FoE				
			DIB				
Smith et al. [70]	2021	IGBT	Array circular coils	-	-	No	Not discussed
		Multichannel					
Sorkhabi et al. [71]	2022	IGBT	FoE	-	-	No	Rehabilitation
		H-bridge	sham				
Zeng et al. [72]	2022	IGBT	-	-	-	No	Not discussed
		Modular					
		Cascade					

## Data Availability

The data presented in this study are openly available in FigShare at https://doi.org/10.6084/m9.figshare.20416101, reference number [141].

## Abbreviations

The following abbreviations are used in this manuscript:

BEM	Boundary Element Method
BEM-FMM	Boundary Element Method with Fast Multipole Method
DAQ	Data Acquisition
DBU	Downward-Bending U-shape
DIB	Double-Inverted Butterfly (coil)
dsPIC	digital signal Programmable Intelligent Computer
FDM	Finite Differences Method
FEM	Finite Element Method
FoE	Figure of Eight (coil)
FPGA	Field-Programmable Gate Array
FBM	Functional Brain Mapping
IBEM	Inverse Boundary Element Method
IGBT	Insulated Gate Bipolar Transistors
IR	Infrared
LFMS	Low-Field Magnetic Stimulation
MEP	Motor Evoked Potential
MOSFET	Metal–Oxide–Semiconductor Field-Effect Transistor
MRI	Magnetic Resonance Imaging
fMRI	functional Magnetic Resonance Imaging
NeNa	Neural Navigator
PET	Positron Emission Tomography
PRISMA	Preferred Reporting Items for Systematic Reviews and Meta-Analyses
ROI	Region of Interest
SCR	Silicon-Controlled Rectifier
SLR	Systematic Literature Review
TBS	Theta-Burst Stimulation
TES	Transcranial Electric Stimulation
TMS	Transcranial Magnetic Stimulation
dTMS	deep Transcranial Magnetic Stimulation
rTMS	repetitive Transcranial Magnetic Stimulation
UBU	Upward-Bending U-shape
WCS	Water-Cooling Stimulation (coil)
